# Droplet volume variability as a critical factor for accuracy of absolute quantification using droplet digital PCR

**DOI:** 10.1007/s00216-017-0625-y

**Published:** 2017-09-18

**Authors:** Alexandra Bogožalec Košir, Carla Divieto, Jernej Pavšič, Stefano Pavarelli, David Dobnik, Tanja Dreo, Roberto Bellotti, Maria Paola Sassi, Jana Žel

**Affiliations:** 1 Department of Biotechnology and Systems Biology, National Institute of Biology, Večna pot 111, 1000 Ljubljana, Slovenia; 2 Jožef Stefan International Postgraduate School, Jamova 39, 1000 Ljubljana, Slovenia; 3 Instituto Nazionale di Ricerca Metrologica, Strada delle Cacce, 91-10135 Turin, Italy

**Keywords:** Droplet digital PCR, Droplet volume, DNA quantification, Optical microscopy imaging

## Abstract

Accurate and precise nucleic-acid quantification is crucial for clinical and diagnostic decisions, as overestimation or underestimation can lead to misguided treatment of a disease or incorrect labelling of the products. Digital PCR is one of the best tools for absolute nucleic-acid copy-number determination. However, digital PCR needs to be well characterised in terms of accuracy and sources of uncertainty. With droplet digital PCR, discrepancies between the droplet volume assigned by the manufacturer and measured by independent laboratories have already been shown in previous studies. In the present study, we report on the results of an inter-laboratory comparison of different methods for droplet volume determination that is based on optical microscopy imaging and is traceable to the International System of Units. This comparison was conducted on the same DNA material, with the examination of the influence of parameters such as droplet generators, supermixes, operators, inter-cartridge and intra-cartridge variability, and droplet measuring protocol. The mean droplet volume was measured using a QX200™ AutoDG™ Droplet Digital™ PCR system and two QX100™ Droplet Digital™ PCR systems. The data show significant volume differences between these two systems, as well as significant differences in volume when different supermixes are used. We also show that both of these droplet generator systems produce droplets with significantly lower droplet volumes (13.1%, 15.9%, respectively) than stated by the manufacturer and previously measured by other laboratories. This indicates that to ensure precise quantification, the droplet volumes should be assessed for each system.

**Electronic supplementary material:**

The online version of this article (10.1007/s00216-017-0625-y) contains supplementary material, which is available to authorized users.

## Introduction

Accurate and precise nucleic-acid quantification is fundamental in many fields, from basic research to molecular diagnostics, and in preclinical and clinical research and industrial processes. Absolute DNA quantification allows the reduction of measurement bias between laboratories, which is essential for target DNA measurements in medicine, viral load analysis in diagnostics, and microbial quantification in microbiology. Currently, the most used method for DNA quantification is real-time polymerase chain reaction, which depends on the standard curve approach for quantification. However, the choice of the standard can in some fields be arbitrary and is often not uniform among laboratories [1]. Absolute quantification of nucleic acids without the need of a standard curve can be achieved with digital PCR (dPCR). Taking into account the high potential of absolute quantification by dPCR, this technique represents a good candidate for reference methods for nucleic-acid copy-number determination [1, 2].

Although the concept of dPCR was defined in the 1990s [3, 4], dPCR has gained further popularity in more recent years [1, 5–14], with 442 papers listed in the Scopus database for 2016, compared to 125 in 2012 (Fig. S1 in the Electronic Supplementary Material, ESM). The main characteristic of dPCR is the dispersal of the reaction volume into partitions, either chambers in chamber/microfluidics-based digital PCR, or droplets in droplet digital PCR (ddPCR). The underlying assumptions of dPCR which need to be met include a random distribution of DNA molecules into the partitions and amplification of a single target in a partition [15, 16]. To achieve this, the sample and reaction preparation need to be carefully controlled, and the dPCR needs to be well characterised in terms of accuracy and sources of uncertainty.

In dPCR, the copy number concentration in the sample is calculated according to Eq. (1),

1 $$ Tc=-\mathit{\ln}\left(1-\frac{P}{R}\right)\times \left(\frac{1}{Vd}\right)\times D $$

where *Tc* is the mean target concentration (copies/μL), *P* is the number of positive partitions, and *R* is the number of analysed partitions. The copy number concentration depends also on the partition volume (*Vd*) and the dilution factor of the original solution in the PCR reaction (*D*). Consequently, the accuracy of absolute quantification is strongly dependent on correct determination of the partition volume, which is the droplet volume for ddPCR, and on the number of partitions. The confidence of measurements can thus be enhanced by accurate determination of the partition volume and by raising the number of reactions (i.e. the number of partitions) for any one sample.. If the droplet volume varies among reactions or droplet generators, the copy number can be either underestimated or overestimated, depending on whether the droplet volume is overestimated or underestimated, respectively. Additionally, as was clearly illustrated by Huggett et al. [5], if there is partition volume variability within one reaction, this can lead to a smaller number of partitions with a positive signal [5]. Hence, if partition volume variability is not considered in the calculation of DNA copy numbers, bias in the estimated number of copies per reaction can be attributed to incorrect droplet volume assessment and to non-homogeneous distribution of molecules among partitions.

Discrepancies between droplet volumes assigned by the manufacturer and measured by independent laboratories have been shown in previous studies [17–19], where the droplet volumes were measured for QX100™ Droplet Digital™ PCR systems (hereafter indicated as a DG8 manual droplet generator; Bio-Rad). The QuantaSoft™ software version 1.3.2.0 used the pre-set volume of 0.91 nL [18]. Further on, droplet volumes were measured independently at the Australian National Measurement Institute (NMIA) and by Bio-Rad. NMIA measured droplets in 16 wells across five separate cartridges and determined a volume of 0.868 nL with a relative standard deviation of the mean inter-well droplet volume of 2.8%. The Bio-Rad measure for droplets generated from the eight-channel droplet generator cartridges was 0.89 nL [17], which showed that the droplet volume was smaller than previously believed. Additionally, even lower droplet volume, measured as 0.834 nL, was determined by Corbisier et al. [18]. It is prudent here to point out that measurements at the NMIA were conducted on an early access beta-prototype ddPCR system; thus, it is not necessary that this system generated droplets of same volume as current versions of QX100™ Droplet Digital™ PCR and QX200™ AutoDG™ Droplet Digital™ PCR system. And although the same droplet volume was pre-set regardless of the droplet generator used, a direct comparison cannot be made.

Abovementioned measures by Corbisier et al. [18] were performed on a discontinued series of cartridges (DG8-186-3008). More recently, a new series of cartridges (DG8-186-4008) became available on the market. At the same time the QuantaSoft™ software was updated and the new version had a new droplet volume of 0.85 nL incorporated in the calculations. Although there have been assessments of supermix effects on droplet volume (ddPCR™ Supermix™ for Probes [17–19]; ddPCR™ Supermix™ for Probes (no dUTP) [19]; QX200™ ddPCR™ Eva Green™ Supermix™ [18]), all of the studies to date have been focused only on the DG8 manual droplet generator and to the best of our knowledge no study was conducted on the new series of cartridges.

In the present study, the partition volume was considered in an inter-laboratory comparison with different methods for volume determination. A comparison of droplet volume measurements was conducted on the same DNA material, while taking into account further parameters, including droplet generator, supermix type, laboratory operator, inter-cartridge and intra-cartridge variability, and droplet measuring protocol. Mean droplet volumes were measured for a QX200™ AutoDG™ Droplet Digital™ PCR system (hereafter indicated as DG32 automated droplet generator) at the National Institute of Biology (NIB; Ljubljana, Slovenia) and for two DG8 manual droplet generators, one at NIB and the other at the Italian National Metrology Institute - Instituto Nazionale di Ricerca Metrologica (INRiM; Turin, Italy).

## Materials and methods

### Test material, and methods

The American Oil Chemists’ Society 0707-B4 certified reference material that contains 99.99 mass/mass% of A2704-12 (ACS-GMØØ5-3) genetically modified (GM) soybean was used as the DNA sample. A duplex assay that targets the GM event (A2704-12) and the endogene (*Le1*) was used. The primer and probe sequences were taken from GMOMETHODS, a European Union database of reference methods for analysis of genetically modified organisms (GMOs) (http://gmo-crl.jrc.ec.europa.eu/gmomethods/), with the entry names of QT-EVE-GM-004 and QT-TAX-GM-002 for A2704-12 and lectin, respectively. To exclude the sample as a cause of droplet volume variation, the same sample was used for the measurement of all droplets at NIB and INRiM.

### Droplet volume determination

#### Experimental design

The volume of the droplets was calculated by measuring the individual droplet diameters. Droplet volume was determined for three different experimental set-ups: (1) DG8 manual droplet generator (Bio-Rad, Pleasanton, CA, USA) and ddPCR Supermix™ for probes (no dUTP) (Bio-Rad, Pleasanton, CA, USA); (2) DG32 automated droplet generator (Bio-Rad, Pleasanton, CA, USA) and ddPCR Supermix™ for probes (no dUTP); and (3) DG8 manual droplet generator and QX200 ddPCR EvaGreen™ Supermix™ (Bio-Rad, Pleasanton, CA, USA) (Fig. S2, see ESM). For experimental set-ups (1) and (2), three cartridges were analysed over three consecutive days, and for experimental set-up (3), three cartridges were analysed on the same day. Experimental set-up (1) was carried out using two different machines (one at NIB, one at INRiM), to determine the variability of the DG8 droplet generators. The same reaction mix containing supermix (either ddPCR Supermix™ for probes (no dUTP) or EvaGreen™ Supermix™), DNA, primers and probe (200 nM final concentration) was used for all set-ups. The same Lot N° of DG8 cartridges (000036132) was used in experimental set-ups (1) and (3). For experimental set-ups (1) and (2), six wells in each cartridge were randomly selected, and for experimental set-up (3), four wells in each cartridge were randomly selected.

#### Optical microscopy

Droplet preparation and optical microscopy imaging were performed on the same day. After droplet generation, 10 μL of droplets was transferred into 24-well polystyrene cell culture plates (Corning Inc., Corning, NY, USA). The droplets were left to form a uniform monolayer by positioning the plates at an angle of approximately 45° to the horizontal axis for a few seconds. The droplets for each of the experimental set-ups were imaged using an optical microscope (NIB: Nikon Eclipse™ T*i*-E; INRiM: Zeiss Axio Observer™ Z1), with monochromatic, high-resolution digital CCD cameras (NIB: Clara™, Andor Technology; INRiM: digital Olympus XM10™). All of the images were recorded in bright field under uniform illumination and × 100 apparent magnification. Digital images were captured using Nikon NIS-Elements™ Advanced Research software at NIB, and the Olympus cellSens™ microscope imaging software at INRiM. The droplets were imaged immediately after their formation, to avoid evaporation and shrinking (Fig. S3, see ESM). An average of six images per well was acquired, and 86 to 284 droplets were measured for each well. For each experimental set-up, the following total number of droplets were measured: set-up (1) at NIB, 3091 droplets; set-up (1) at INRiM, 3567 droplets; set-up (2), 3533 droplets; and set-up (3), 2140 droplets (Fig. S2, see ESM).

#### Optical profilometer

To ensure traceability to the International System of Units (SI), an optical profilometer (Plμ 2300™, Sensofar) and software for image analysis were used as the reference method for droplet diameter measurements. The software was validated within the EURAMET iMERA-Plus “Traceable Characterisation of Nanoparticles” project [20]. The x–y measurement system was traceable to the SI, so the diameter measurement was traceable to the SI. The optical profilometer and the entire reference method are described in Methods S1 in the ESM.

#### Image analysis and validation

The Fiji™ software [21], a distribution of ImageJ™ [22, 23], was used to analyse the digital images of the droplets. Two droplet measurement protocols were compared, both of which used the Fiji™ software. The droplet measurement protocol referred to as ‘manual’ was developed at INRiM and is described in Methods S2 in the ESM. The traceability of the diameter measurements to the SI unit of ‘metre’ was assured by comparison of the manual and automated droplet measurements with the optical profilometer-based method, which was used as a reference method, as described in Methods S1 (see ESM). The ‘automatic’ droplet measurement protocol was adapted from Pinheiro et al. [17], and is described in Methods S3 in the ESM. The automatic droplet measurements were used to compare the data from different operators, one at NIB and one at INRiM, and to eliminate the possibility of any *between-operators effect*. For this, images obtained from experimental set-up (1) for day 1 at INRiM were analysed independently by two different operators. All of the sequential image analysis was performed with the automatic droplet measurement protocol, at both NIB and INRiM.

### Droplet digital PCR

#### PCR conditions

A 20-μL reaction mixture was prepared comprising of 10 μL ddPCR Supermix™ for probes (no dUTP) (Bio-Rad, Pleasanton, CA, USA), 6 μL primers and probe mix, and 4 μL DNA. The final concentration of both primers and probe was 200 nM. For each droplet generator, 24 ddPCR reactions were performed on three consecutive days, which included six negative template controls. The amplification conditions were 10 min DNA polymerase activation at 95 °C, followed by 40 cycles of a two-step thermal profile of 30 s at 94 °C for denaturation, and 60 s at 60 °C for annealing and extension, followed by a final hold of 10 min at 98 °C for droplet stabilisation, and cooling to 4 °C. A thermal cycler (T100™; Bio-Rad, Pleasanton, CA, USA) was used, and the temperature ramp rate was set to 2.5 °C/s, with the lid heated to 105 °C, according to the Bio-Rad recommendations.

#### Data analysis

After the thermal cycling, the plates were transferred to a droplet reader (QX100™; Bio-Rad, Pleasanton, CA, USA). The software package provided with the ddPCR system was used for data acquisition (QuantaSoft™ 1.6.6.0320; Bio-Rad). The rejection criterium for the exclusion of a reaction from subsequent analysis was a low number of droplets measured (< 10,000 per 20 μL PCR). The data from the ddPCR are given in target copies/μL reaction.

### Analysis of results

#### Statistical analysis of droplet volume

To evaluate the differences between the droplet volumes calculated for each individual droplet generator, single-factor analysis of variance (ANOVA) and Tukey’s tests were used in R studio™, version 1.0.136 [24]. Outliers were determined for each experimental set-up independently, using Grubbs tests (R studio™, ‘outliers’ package), and were excluded from further analysis.

The bottom-up approach was used to determine the expanded measurement uncertainty [25]. A combined measurement uncertainty *u*_*c*_ was calculated according to Eq. (2) [26],

2 $$ {u}_c=\sqrt{{u_r}^2+{u_{ip}}^2\ } $$

where *u*_*r*_ is the uncertainty associated with the repeatability, and *u*_*ip*_ is the uncertainty associated with the intermediate precision. *u*_*r*_ and *u*_*ip*_ (*u*_*ip1*_ or *u*_*ip2*_) were calculated according to Eqs. (3), (4) and (5):

3 $$ {u}_{\mathrm{r}}=\frac{\sqrt{MS_{\mathrm{within}}}\ }{\sqrt{n}} $$

4 $$ {u}_{ip1}=\sqrt{\frac{MS_{\mathrm{between}}-{MS}_{\mathrm{within}}}{n\times N}} $$

5 $$ {u}_{ip2}=\frac{\sqrt{\frac{MS_{\mathrm{within}}\ }{n}}\times \sqrt[4]{\frac{2}{N\times \left(n-1\right)}}}{\sqrt{N}} $$

where *n* is the number of independent replicates per experiment, *N* is the number of experiments performed on one platform, *MS*_within_ is the mean square value within groups, and *MS*_between_ is the mean square value between groups. Both of these mean squares were calculated using ANOVA in Microsoft Excel™ 2016, with all of the measurements taken into account for each droplet generator. If *MS*_between_ > *MS*_within_, Eq. (4) was used to calculate the intermediate precision, otherwise Eq. (5) was used. A coverage factor (*k*) of 2.1 was chosen at the 95% level of confidence, based on the degrees of freedom, and was applied to obtain the expanded measurement uncertainty.

## Results and discussion

Digital PCR is becoming more and more accepted as a tool for absolute quantification, and thus there is the need to assure the accuracy of its copy-number determination. For this, it is critical to determine the droplet volume, as this is one of the key factors for accuracy of absolute quantification in ddPCR. Earlier studies have shown that differences in droplet volumes can arise due to different supermix types [18, 19], with potential differences also seen for different droplet generator [18]. For the purpose of an inter-laboratory comparison of how different factors affect droplet volume, the mean droplet volume was measured independently both at NIB and INRiM using the same droplet measurement protocol. For this purpose, three experimental set-ups were prepared: (1) DG8 manual droplet generator and ddPCR Supermix™ for probes (no dUTP); (2) DG32 automated droplet generator and ddPCR Supermix™ for probes (no dUTP); and (3) DG8 manual droplet generator and QX200 ddPCR™ EvaGreen™ Supermix™ (Fig. S2 in the ESM).

### Droplet diameter and droplet volume measurements are traceable and accurate

To calculate the droplet volume, measurements of droplet diameters were obtained using microscopic imaging. The volume measurement must be metrologically traceable [27]. To determine the SI traceability and the accuracy of the droplet imaging protocols for droplet diameter measurement, the diameters obtained by the manual and automatic droplet measurement protocols were compared with results obtained using an optical profilometer-based reference method (Tables S1 and S2 in the ESM). The reference method and the traceability process are fully described in Methods S1 in the ESM. The accuracy of the reference method was higher than 0.8%, while the variability of the droplet diameter measurement was 2% (*k* = 1) (Table S1 in the ESM). Data from the comparison between the reference method and each of the manual and automatic droplet measurement protocols showed that the results were comparable with the reference method within a bias of 1.3 and 0.9%, respectively (Table S2 in the ESM). The optical-microscopy-based manual and automatic droplet measurement protocols can thus be considered traceable to the SI through comparison with the reference method.

A comparison between the manual and automatic droplet measurement protocols was used on three sub-sets of measurements (sub-sets of wells 1 and 2 in experimental set-up (1) for day 1 at NIB). Data from these three sub-sets showed that the droplet measurement protocols give comparable results, with the maximum of 1.9% bias, and comparable relative standard deviations (RSDs) (Table S3 in the ESM). As the automatic droplet measurement protocol is quicker, further analysis was conducted using this automatic droplet measurement only. Additionally, a validation of the image analysis used for the automatic droplet measurement protocol was performed, to eliminate the possibility of differences in volume due to different operators. No *between-operators effect* was observed: in both cases, the mean determined droplet volume was 0.708 nL, with standard deviations of 0.025 and 0.022 for NIB and INRiM, respectively (Table S4 in the ESM). The bias between the droplet volumes was − 0.02%, and the bias between the mean area equivalent diameters was − 0.01%. As different operators did not have any effects on the droplet volume measurements, comparisons were then possible for the droplet volumes measured in the different laboratories following the same droplet measurement protocol.

### Droplet generators have an effect on droplet volume

The mean droplet volume for experimental set-ups (1) and (2) was measured for each of the analysis days (Table 1). The mean volume of the droplets generated by the DG8 manual droplet generator was 0.715 nL at NIB and 0.720 nL at INRiM (Table 1). No significant differences were observed between the droplet volumes for each of the DG8 droplet generators (*p* value, 0.939). The RSDs for the inter-well mean droplet volumes were 5.46 and 4.37% for the DG8 manual droplet generator at NIB and the DG8 manual droplet generator at INRiM, respectively. The mean droplet volume between the two laboratories was calculated as 0.718 nL, which is considerably lower than the 0.850 nL that is used in the QuantaSoft software™ from version 1.6.6.0320 onwards. The measured volume is also lower than the 0.834 nL determined by Corbisier et al. [18] and the volumes measured at the NMIA, from where volumes of 0.868 [17] and 0.833 [18] nL were reported. However, a direct comparison cannot be made, as Corbisier et al. used a different supermix (ddPCR Supermix™ for probes) and different cartridges, and as mentioned above, droplets were generated using a beta version of the droplet generator at NMIA. Nevertheless, the difference in droplet volume shows that a fixed volume used in QuantaSoft software™ may cause bias in copy-number determination. The droplet volumes determined in the present study are closer to the values measured at the National Institute of Standards and Technology (USA) in 2016 [19], where the mean droplet volume for ddPCR Supermix™ for probes (no dUTP) was 0.780 and 0.767 nL using diluted and concentrated methods, respectively. The principle of the diluted method is the same as that used in the present study.

**Table 1:** Comparisons of the mean area equivalent diameters and mean droplet volumes (±standard deviation) produced by different droplet generators for the three individual analysis days

Droplet generator	Measurement day	Area equivalent diameter (μm)	Droplet volume (nL)	Expanded measurement uncertainty (nL)
DG8 NIB	1	110.97 ± 2.00	0.716 ± 0.04	
	2	110.23 ± 1.89	0.702 ± 0.03	
	3	111.33 ± 2.08	0.723 ± 0.04	
	Mean*	110.89 ± 2.05	0.715 ± 0.04^a^	0.014
DG8 INRiM	1	110.58 ± 1.17	0.708 ± 0.02	
	2	111.47 ± 1.46	0.725 ± 0.03	
	3	111.50 ± 2.18	0.726 ± 0.04	
	Mean*	111.28 ± 1.73	0.720 ± 0.03^a^	0.013
DG32	1	112.73 ± 1.24	0.750 ± 0.02	
	2	112.25 ± 2.32	0.741 ± 0.04	
	3	111.52 ± 2.65	0.727 ± 0.05	
	Mean*	112.12 ± 2.28	0.739 ± 0.04^b^	0.016

Data with different superscript letters are significantly different (*p* < 0.05; ANOVA)

*Means are calculated from all of the accepted droplets during all 3 days of measurement

**Fig. 1:**
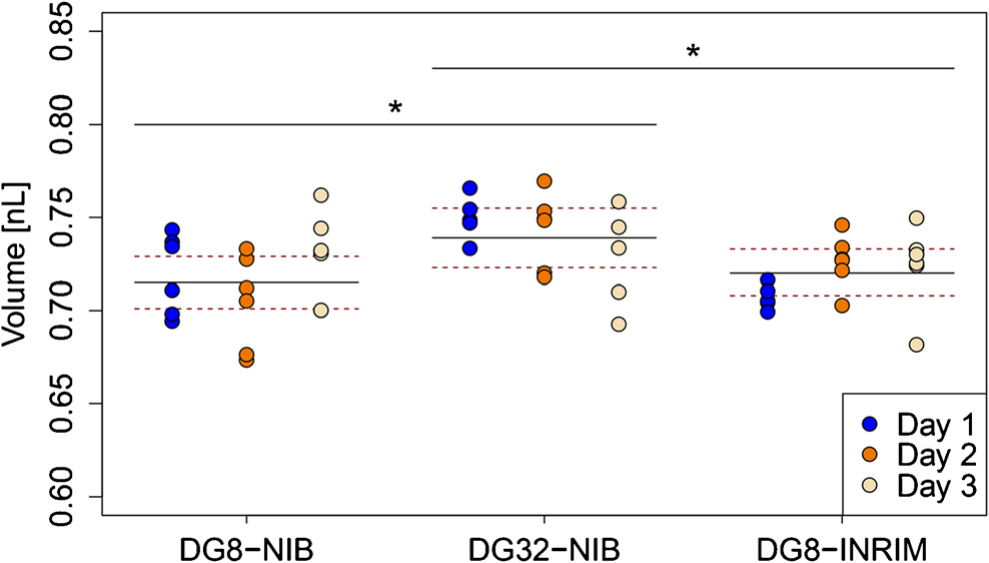
Inter-cartridge variability and differences between the droplet generators. The inter-cartridge variability is shown by the dispersal of the dots that represent mean droplet volumes of one well in a cartridge on that day. The full black line indicates mean droplet volume and the dotted red lines indicate expanded measurement uncertainty. The droplet volumes between the DG8 (manual) and DG32 (automatic) droplet generators are significantly different (*p < 0.05; ANOVA), while there are no significant differences between the two DG8 droplet generators

The mean droplet volume measured for the DG32 droplet generator was 0.739 nL, with RSD of 5.84% (Table 1). This was significantly different to the DG8 results for both NIB and INRiM (*p* values, 0.011, 0.029, respectively) (Fig. 1). Although the DG32 droplet generator volume was larger than the volume measured for both DG8 manual droplet generators, the volume was smaller than those reported in previous studies [17–19].

The same Lot N° of cartridges was tested with each of the DG8 manual droplet generators, which indicated that the DG8 manual droplet generator on its own does not have any effect on droplet volume. However, the droplet volume for the DG32 automated droplet generator was significantly higher than that of DG8. This can lead to the conclusion that droplet volumes can vary depending to the type of droplet generators (i.e. DG8 and DG32 droplet generators).

### Intra-cartridge and inter-cartridge variability is not significant

The intra-cartridge and inter-cartridge droplet volume variabilities were determined for all of the experimental set-ups. Grubbs tests showed that none of the wells can be considered as an outlier for the DG8 droplet generator at NIB, while there was one outlier (day 1) for the DG8 droplet generator at INRiM and two outliers for the DG32 droplet generator (days 1, 3) (Table S5 in the ESM). The use of ANOVA followed by Tukey’s tests showed no significant intra-cartridge (*p* values, 0.206 to 0.761) and inter-cartridge (*p* values, 0.151 to 0.252) effects. Thus, potential effects that a specific position in a cartridge (specific well) would have on copy number determination for a sample can be excluded as a significant uncertainty source that affects the accuracy of ddPCR in the present study.

### Supermix™ has a significant influence on droplet volume

**Fig. 2:**
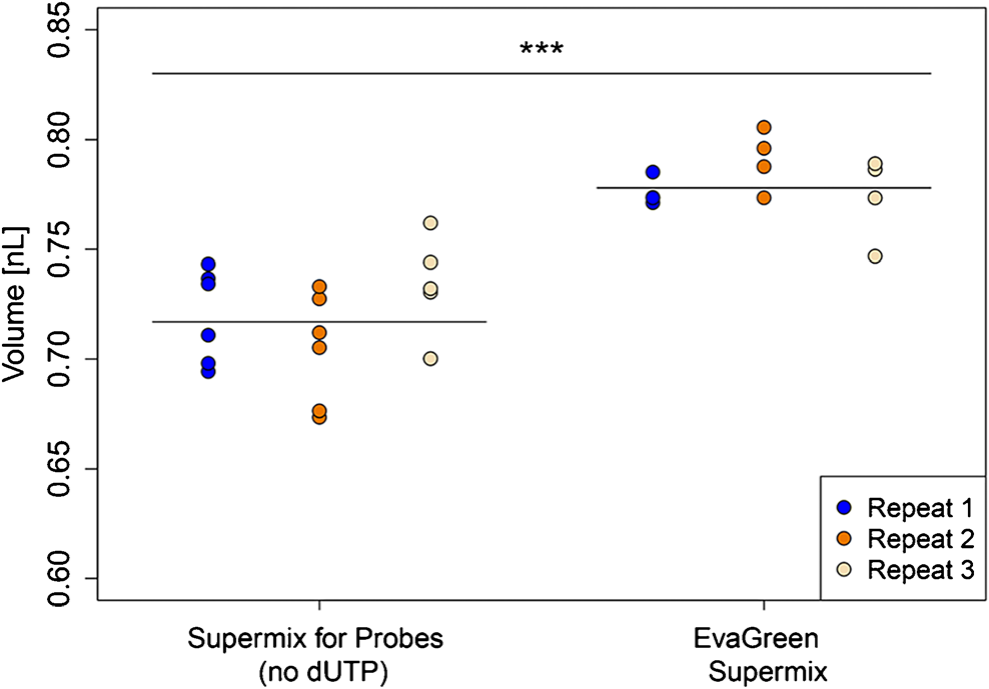
Comparison of droplet volumes between the two different supermixes, Supermix™ for probes (no dUTP) and EvaGreen™ Supermix™. The inter-cartridge variability is shown by the dispersal of the dots that represent mean droplet volumes of one well in a cartridge for each of the three repeats. The full black line indicates mean droplet volume. The droplet volumes between the different supermixes are significantly different (***p < 0.001; ANOVA)

The mean droplet volume measured for the three cartridges using EvaGreen™ Supermix™ was 0.778 nL, with RSD of 3.08% (Table S6 in the ESM). With the same DNA sample and the same Lot N° of the cartridges used in the comparisons of different droplet generators, the droplet volume was significantly larger when EvaGreen™ Supermix™ was used, compared to the droplet volume measured when using Supermix™ for Probes (*p* value, < 0.01) (Fig. 2). The droplet volume is thus not only dependent upon the type of droplet generator, but also on the type of supermix.

### Differences in copy-number determination are correlated with droplet volume

Copy numbers were determined for one dilution (100×) of the certified reference material that contained 99.9% A2704-12 GM soybean. The copy numbers were determined over three consecutive days to ensure sufficient numbers of test replicates (*n* = 18), repeatability and reproducibility, to be consistent with the ‘Definition of Minimum Performance Requirements for Analytical Methods of GMO Testing’ and the ‘Minimum Performance Parameters and Validation Aspects on PCR Amplification’ defined within the Decathlon Project [28, 29]. Each day one cartridge was prepared, with six replicates of reference DNA and two replicates of negative template control. Negative control wells were positioned randomly. Droplets were prepared with both the DG8 and DG32 droplet generators. The data were analysed using both the droplet volume of 0.85 nL, as specified by the manufacturer, and the volumes measured within this study, 0.715 nL for the DG8 manual droplet generator, and 0.739 nL for the DG32 automated droplet generator.

The relative repeatability standard deviation (RSDr) for the copy number, which provides the precision of the method, was 5.73% for A2704-12 and 5.37% for *Le1* using the DG8 droplet generator, and 5.69% for A2704-12 and 5.39% for *Le1* using the DG32 droplet generator. These are well below the minimum performance criteria of ≤ 25% (Tables S7 and S8 in the ESM) [28, 29]. The mean copy number for all of the 18 measurements with the DG8 manual droplet generator was compared to the measurements obtained with the DG32 automated droplet generator, and the bias between these copy-numbers was calculated (Table 2).

**Table 2:** Mean estimated copy numbers of the A2704-12 and *Le1* targets in the certified reference material, and the expanded measurement uncertainty, and bias between copy numbers obtained with the DG8 and DG32 droplet generators, for the fixed (0.85 nL) and measured (0.715 nL, 0.739 nL, for DG8, DG32, respectively) droplet volumes

Droplet volume (nL)	Target	DG8		DG32		Bias copies/μL DG8/DG32 (%)
		Copies/μL	Measurement uncertainty	Copies/μL	Measurement uncertainty	
0.85	A2704-12	115,351	6034	114,131	5934	1.07
	*Le1*	119,230	5855	117,369	5789	1.59
0.715/0.739	A2704-12	137,131	7174	131,274	6826	4.46
	*Le1*	141,742	6961	134,998	6659	5.00

The relative reproducibility standard deviation (RSD_R_) was calculated, which is defined as the relative standard deviation of the results obtained with the same method on the same test items using different equipment and with different operators. For a method to be compliant with the minimum performance parameters, the RSD_R_ should be ≤ 35% [28, 29]. The RSD_R_ between the DG8 manual droplet generator and the DG32 automated droplet generator using the fixed droplet volume was 5.65% for A2704-12 and 5.37% for *Le1*, and using the measured droplet volume was 6.05% for A2704-12 and 5.86% for *Le1* (Table S9 in the ESM). Thus, although the ANOVA shows that there are statistically significant differences between the droplet volumes for the DG8 manual droplet generator and the DG32 automated droplet generator, the difference has less of an effect on the copy number than the overall difference in droplet volume between 0.85 nL and the measured volume. If the measured droplet volumes are used, the bias in the DNA copy number calculated for the DG8 and DG32 systems are 15.9 and 13.1%, respectively (Table S10 in the ESM). This bias is within the limits set in the currently accepted minimum performance parameters [28, 29]. The bias due to the droplet volume measurements can be taken into account as components of the combined uncertainty of the DNA copy number measured by ddPCR.

In the field of GMO diagnostics, apart from copy number, the GM% (percentage of genetically modified ingredient) is also critical, and is calculated as the ratio between GM and endogene copy number. As the droplet volume affects both of the PCR amplicons, the GMO (A2704-12) and endogene (*Le1*), there is no difference in GM% when comparing fixed and measured droplet volumes. In both cases, the mean GM% with expanded measurement uncertainty was 96.76 ± 2.29 and 97.24 ± 1.76 for the DG8 manual droplet generator and the DG32 automated droplet generator, respectively. Unlike the case of copy number (Table 2), although the expanded measurement uncertainty for the DG8 manual droplet generator was still very low, it was higher than that of the DG32 automated droplet generator. However, the bias between the GM% of the DG8 manual droplet generator and the DG32 automated droplet generator was − 0.5%, which is again well within the limits set in the minimum performance parameters [28, 29].

## Conclusions

This study shows the importance of correct droplet volume determination for absolute quantification of nucleic acids by ddPCR, and highlights the factors that can influence the droplet volume. The droplet volume was based on the measurement of the droplet diameter, from which the droplet volume was calculated, assuming the droplets are (nearly) perfect spheres. To make sure the droplet measurements were reliable, and thus provided accurate results, a reference method traceable to the SI was used for diameter measurements and compared with the two methods used in the present study. This is a calibrated profilometer that is directly traceable to the SI, and was validated in an inter-laboratory comparison study among national metrology institutes. This allowed metrological validation of the methods used, which assures traceable results, and provides a verifiable link to the relevant SI unit—‘metre’. Additionally, the reference method (using the optical profilometer and the unit cell software method) was applied to measure the minor and major axes of the droplets and an average difference between the axes length of 1.8% was obtained. Thus, the assumption that droplets are (nearly) perfect spheres was taken. The microscopy imaging procedure was well characterised when the effects of the dimension calibration of the microscopes were taken into account. The results of the inter-laboratory comparisons between NIB and INRiM were highly comparable, and revealed a droplet volume that was significantly smaller than the previous values measured and used by the manufacturer. The results from this study show that, if the droplet volume is not accurately measured, this can be a source of bias that will influence the DNA copy number measured using this ddPCR platform. Indeed, several factors can affect the droplet volume, which confirms some previous data and gives some new insights into the DG32 system. The mean droplet volumes measured in this inter-laboratory study for experimental set-ups (1), (2) and (3) were 0.715, 0.739 and 0.778 nL, respectively. These are significantly lower than the 0.85 nL that is used in version 1.7.4 of the QuantaSoft™ software from Bio-Rad. The application of an incorrect volume will lead to an underestimation or overestimation of the copy number, which can in turn have effects on clinical and diagnostic decisions, and potentially lead to incorrect labelling of a product or misguided treatment or diagnosis. It should also be taken into account that when transferring the method from EvaGreen™ chemistry to Taq-Man™ chemistry, the supermix used needs to be changed, and that the droplet volume needs to be considered when assessing the copy number. Thus, to accurately determine copy number, the droplet volume should be measured for all possible droplet generators and supermixes used in a laboratory. However, this is time consuming and impractical for the majority of laboratories. Therefore, laboratories where the droplet volume cannot be assessed, and particularly for laboratories with applications in clinical, diagnostic and food-related fields, and in research fields, the measurement uncertainty of the data should be expanded to encompass any influences of droplet volume variability.

## Electronic supplementary material

ESM 1 (PDF 1930 kb)
