# Supplementary material for: Droplet volume variability as a critical factor for accuracy of absolute quantification using droplet digital PCR
Source: Anal Bioanal Chem. 2017 Sep 18;409(28):6689–97. doi: 10.1007/s00216-017-0625-y (PMC5670190; doi:10.1007/s00216-017-0625-y)
Supplement: Supplementary file 1 — (PDF 1930 kb) [file 216_2017_625_MOESM1_ESM.pdf]

Analytical and Bioanalytical Chemistry

Electronic Supplementary Material

Droplet volume variability as a critical factor for accuracy of absolute quantification using droplet digital PCR

Alexandra Bogožalec Košir, Carla Divieto, Jernej Pavšič, Stefano Pavarelli, David Dobnik, Tanja Dreo, Roberto Bellotti, Maria Paola Sassi, Jana Žel

Table of contents:

Fig S1 Papers reporting on digital PCR (blue) and droplet digital PCR (green) from 1986 to 2016. 3
Fig S2 Description of the experimental set-ups........4
Fig S3 Comparison of droplet imaged immediately after generation (a) and after being left on the plate for one hour (b)........5
Table S1 Assigned value of droplet diameter measure by optical profilometer. 6
Table S2 Comparison of manual and automatic droplet measurement protocols with the assigned value gained by optical profilometer. 7
Table S3 Droplet volumes statistics measured with the manual and automatic protocol for the three datasets. 8
Table S4 Validation of image analysis used for the automatic droplet measurements. 9
Table S5 Mean droplet volumes of each individual well, for set-ups (1) and (2), including outliers (grey), for the ddPCR Supermix^{TM} for probes (no dUTP)........10
Table S7 Repeatability and intermediate precision for copy numbers obtained with the DG8 droplet generator for both fixed and measured droplet volumes. 12
Table S8 Repeatability and intermediate precision for copy number obtained with the DG32 droplet generator for both fixed and measured droplet volumes. 13
Table S9 Relative reproducibility standard deviation (RSD_R) for fixed (0.85 nL) and measured (0.715 nL, 0.739 nL, for DG8, DG32, respectively) droplet volumes. 14
Table S10 Bias between copy numbers when using fixed (0.85 nL) and measured (0.715 nL, 0.739 nL, for DG8, DG32, respectively) droplet volumes........15
Methods S1: Reference method for droplet diameter measurement 16
Methods S2: Manual image analysis 18
Methods S3: Automatic image analysis using the Fiji^{TM} software........29
References........33

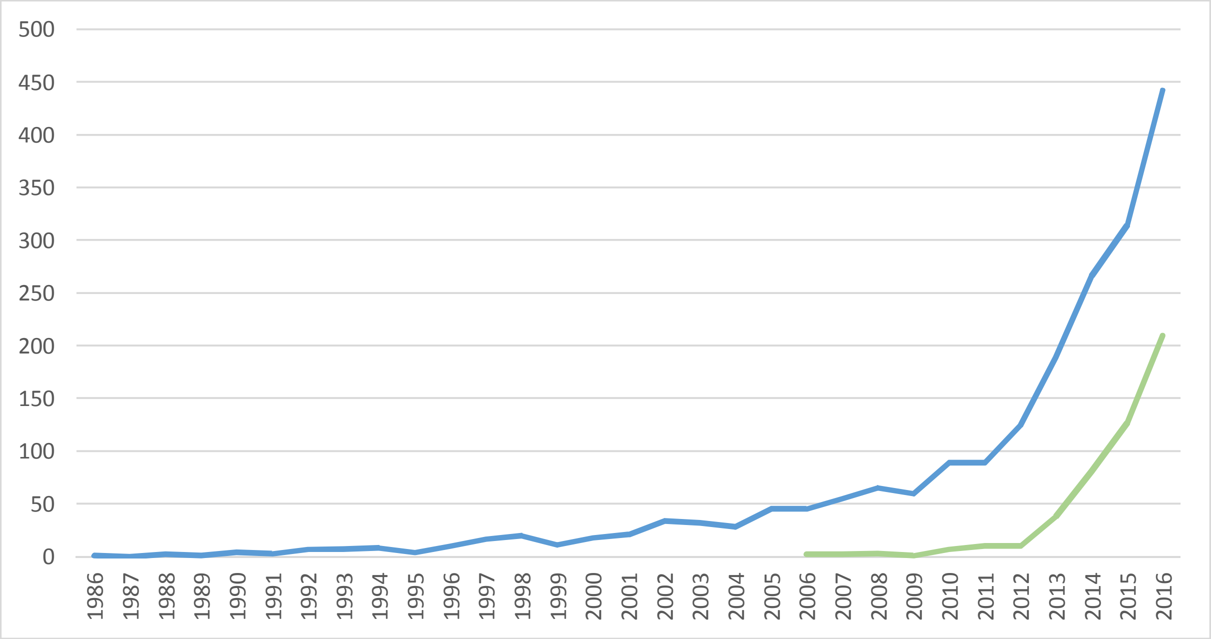

Fig S1 Papers reporting on digital PCR (blue) and droplet digital PCR (green) from 1986 to 2016. Data from Scopus database (accessed 1 February, 2017). In 2017 there were 44 papers listed for digital PCR in general, and of that 27 for droplet digital PCR, in particular. The number of publications in 2017 for both is likely to exceed the number in 2016. Search key word were digital PCR and digital PCR droplet, for dPCR and ddPCR respectively

	Aparatus	Supermix	Number cartridges/ wells per cartridge/ Droplets measured	
Experiment setup (1)	QX100™ Droplet Digital™ PCR System	ddPCR Supermix for probes (No dUTP)	3/6/3091	3/6/3567
Experiment setup (2)	QX200™ AutoDG™ Droplet Digital™ PCR System	ddPCR Supermix for probes (No dUTP)	3/6/3533	
Experiment setup (3)	QX100™ Droplet Digital™ PCR System	QX200 ddPCR EvaGreen Supermix	3/4/2140	

Fig S2 Description of the experimental set-ups. Operators from NIB and INRiM worked on set-ups (1), (2) and (3) at NIB and operators from INRiM worked on set-up (1) at INRiM. All of the droplets were imaged under an optical microscope and all of the images were recorded in bright field under uniform illumination and 100× apparent magnification

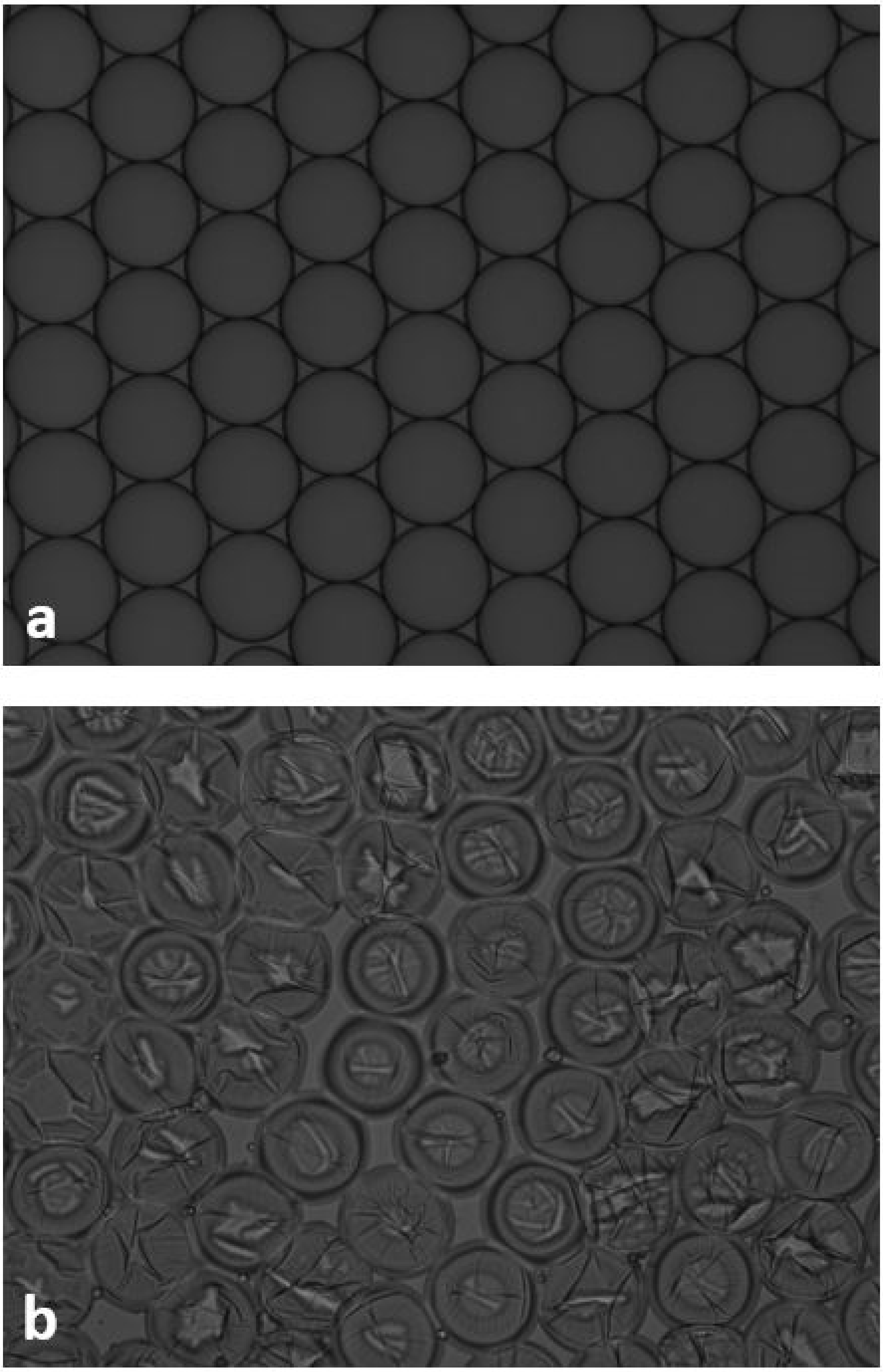

Fig S3 Comparison of droplets imaged immediately after generation (a) and after one hour (b). Droplets left on the plate for one hour were subjected to evaporation. Evaporation causes shrinking and wrinkling, thus these droplets are no longer suitable for imaging

Table S1 Assigned value of droplet diameter measure by optical profilometer. The assigned value was calculated as mean of results from the 3 different methods: based on autocorrelation, unit cell detection and length measurements of lines of packed droplets

**Methods**	**Mean diameter [µm]**	**SD [µm]**	**N° of droplets**	**RSD %**
Autocorrelation	109.68	1.83	303	1.7
Unit cell	109.55	2.36	303	2.2
Line	109.23	2.40	48	2.2
**Reference Value**	**109.48**	**2.20**		**2.0**

Table S2 Comparison of manual and automatic droplet measurement protocols with the assigned value gained by optical profilometer. The comparison of manual and automatic droplet measurement protocols with the reference method was done on 3 different sets of images

Method	Mean diameter [µm]	SD [µm]	N° of droplets	RSD %	Bias to reference %
Reference	109.48	2.20	303	2.0	
Manual	110.91	2.64	136	2.4	1.3
Automatic	110.52	1.77	6175	1.6	0.9

Table S3 Droplet volumes statistics measured with the manual and automatic protocol for the three datasets

Dataset	Manual measurements			Automatic measurements			Mean volume (nL)	Bias (%)
	Volume (nL)	RSD (%)	N° of droplets	Volume (nL)	RSD (%)	N° of droplets		
(1)	0.668	1.06	58	0.669	2.04	44	0.669	-0.15
(2)	0.709	1.54	46	0.723	1.38	41	0.716	-1.94
(3)	0.770	2.19	32	0.777	1.16	37	0.774	-0.90

Table S4 Validation of image analysis used for the automatic droplet measurements

Droplet parameter	Statistic	NIB	INRiM	Bias NIB/ INRiM (%)
Number	(n)	612	1028	
Volume	Mean volume (nL)	0.708	0.708	-0.02
	Standard deviation (nL)	0.025	0.022	
	RSD (%)	3.56	3.05	
Diameter	Mean area equivalent diameter (µm)	110.56	110.58	-0.01
	Standard deviation (µm)	1.39	1.17	
	RSD (%)	1.25	1.06	

Table S5 Mean droplet volumes of each individual well, for set-ups (1) and (2), including outliers (grey), for the ddPCR Supermix^{TM} for probes (no dUTP)

Droplet generator	Set-up	Day 1		Day 2		Day 3	
		Mean droplet volume (nL)	No of analysed droplets	Mean droplet volume (nL)	No of analysed droplets	Mean droplet volume (nL)	No of analysed droplets
DG8	(1) NIB	0.694	229	0.699	133	0.744	155
		0.743	177	0.728	160	0.700	206
		0.711	232	0.733	121	0.762	151
		0.737	165	0.673	130	0.700	284
		0.698	215	0.676	169	0.731	182
		0.734	111	0.705	165	0.732	106
	(1) INRiM	0.654	231	0.734	231	0.682	141
		0.704	149	0.727	200	0.724	186
		0.716	269	0.746	194	0.725	141
		0.705	175	0.727	137	0.750	207
		0.710	244	0.721	140	0.732	220
		0.699	191	0.703	301	0.730	21
DG32	(2) NIB	0.733	117	0.720	236	0.710	241
		0.749	154	0.749	201	0.693	202
		0.747	213	0.753	185	0.745	157
		0.654	189	0.718	228	0.595	265
		0.766	162	0.770	208	0.758	231
		0.754	144	0.748	174	0.733	226

Table S6 Mean area equivalent diameters and droplet volumes ±standard deviation from the EvaGreen^{TM} Supermix^{TM} for the three repeats for the DG8 droplet generator at NIB. Mean droplet volume are calculated from all of the accepted droplets from all four measured wells

Measurement cartridge repeat	Mean area equivalent diameter ±SD (µm)	Mean droplet volume ±SD (nL)
**1**	114.01 ±1.15	0.777 ±0.02
**2**	114.51 ±1.12	0.786 ±0.02
**3**	113.86 ±1.22	0.773 ±0.02
**Overall mean**	**114.11 ±1.20**	**0.778 ±0.02**

Table S7 Repeatability and intermediate precision for copy numbers obtained with the DG8 droplet generator for both fixed and measured droplet volumes

Measurement cartridge repeat	Target	Fixed droplet volume as 0.85 nL		Measured droplet volume as 0.715 nL	
		**Copy number ±SD**	**RSD_r (%)**	**Copy number ±SD**	**RSD_r (%)**
1	A2704-12	113457 ±6597	5.81	134879 ±7843	5.81
	Le1	116242 ±6648	5.72	138190 ±7903	5.72
2	A2704-12	116479 ±4712	4.05	138471 ±5602	4.05
	Le1	121084 ±3938	3.25	143947 ±4682	3.25
3	A2704-12	116117 ±8731	7.52	138041 ±10379	7.52
	Le1	120364 ±7970	6.62	143090 ±9475	6.62
**Overall mean**	**A2704-12**	**115351 ±6609**	**5.73**	**137131 ±7856**	**5.73**
	Le1	**119230 ±6408**	**5.37**	**141742 ±7618**	**5.37**

Table S8 Repeatability and intermediate precision for copy number obtained with the DG32 droplet generator for both fixed and measured droplet volumes

Measurement cartridge repeat	Target	Fixed droplet volume as 0.85 nL		Measured droplet volume as 0.739 nL	
		**Copy number ±SD**	**RSD_r (%)**	**Copy number ±SD**	**RSD_r (%)**
1	A2704-12	110714 ±4676	4.22	127343 ±5379	4.22
	Le1	113916 ±3302	2.90	131026 ±3798	2.90
2	A2704-12	116811 ±3542	3.03	134357 ±4074	3.03
	Le1	120047 ±3405	2.84	138078 ±3917	2.84
3	A2704-12	114868 ±9250	8.05	132122 ±10639	8.05
	Le1	118144 ±9496	8.04	135889 ±10923	8.04
**Overall mean**	**A2704-12**	**114131 ±6491**	**5.69**	**131274 ±7466**	**5.69**
	Le1	**117369 ±6332**	**5.39**	**134998 ±7283**	**5.39**

Table S9 Relative reproducibility standard deviation (RSD_R) for fixed (0.85 nL) and measured (0.715 nL, 0.739 nL, for DG8, DG32, respectively) droplet volumes

**Droplet generator**	**Measurement cartridge repeat**	**Target (copies/µL)**			
		**Fixes droplet volume at 0.85 nL**		**Measured droplet volumes at 0.715 (DG8) nL / 0.739 (DG32) nL**	
		**A2704**	***Le1***	**A2704**	***Le1***
**DG8**	1	101011	104266	120083	123953
		111394	117838	132426	140087
		118441	120009	140804	142668
		118114	123980	140415	147389
		115682	115313	137524	137085
		116100	116046	138021	137957
	2	122316	121267	145410	144164
		110660	119063	131554	141544
		118403	119025	140758	141498
		111417	116338	132453	138304
		120149	127564	142835	151650
		115928	123249	137816	146519
	3	114696	119363	136352	141899
		125549	126380	149254	150242
		121861	125867	144869	149632
		100711	106608	119726	126737
		113907	116706	135414	138742
		119980	127259	142634	151288
**DG32**	1	110570	115338	127178	132662
		108081	114649	124315	131870
		104276	109147	119939	125541
		117429	117478	135067	135124
		109440	110607	125879	127221
		114487	116275	131683	133740
	2	112042	115756	128871	133143
		117026	123916	134603	142528
		122745	123826	141182	142426
		117832	120712	135530	138843
		116253	118979	133714	136850
		114971	117092	132239	134680
	3	96755	101671	111287	116943
		118469	119057	136263	136939
		119933	123335	137947	141861
		122033	129767	140363	149259
		117900	120333	135609	138407
		114120	114699	131261	131927
**SD (copies)**		**6486**	**6349**	**8116**	**8102**
**RSD_R (%)**		**5.65**	**5.37**	**6.05**	**5.86**

Table S10 Bias between copy numbers when using fixed (0.85 nL) and measured (0.715 nL, 0.739 nL, for DG8, DG32, respectively) droplet volumes

**System**	**Target (copies per µL)**			
	**DG8**		**DG32**	
	**A2704-12**	***Le1***	**A2704-12**	***Le1***
Fixes droplet volume at 0.85 nL	115351	119230	114131	117369
Measured droplet volumes at 0.715 (DG8)/ 0.739 (DG32) nL	137131	141742	131274	134998
Bias (%)	-15.88	-15.88	-13.06	-13.06

Methods S1: Reference method for droplet diameter measurement

A reference method for droplet diameter measurement was used to ensure traceability to the International System of Units (SI). The reference method used a combination of a calibrated measurement instrument for image acquisition and two validated image analysis protocols. The calibrated instruments include an optical profilometer (Plμ 2300^{TM}, Sensofar) equipped with a motorised stage for xy movements, a microscope with a 10× objective, and a CCD camera for image acquisition. The profilometer was calibrated with an INRiM two-dimensional standard grating referred to the SI units system. The profilometer is located in a temperature controlled laboratory at INRiM.

The image analysis protocols were validated within the EURAMET iMERA-Plus project "Traceable Characterisation of Nanoparticles" [1] for traceable calibration of nanoparticle sizes at National Metrology Institutes. Within the project, comparison measurements aimed at the validation of methods and uncertainty estimations across six National Metrology Institutes was carried out. All of the measurements were directly traceable to the SI unit 'metre'.

The reference method 'procedure' of the images acquisition by the profilometer and the image analysis was used for the droplet diameter measurements. As a reference method, this is traceable to the SI.

Reference method procedure description

Several images of packed droplets were acquired with an area of 1.27 × 0.95 mm (768 × 576 pixels) using the optical profilometer. The scanning probe image processor (Image Metrology) software was used for image elaboration, to determine the mean diameters of the droplets by lateral measurements using two different methods: (i) as based on autocorrelation and unit cell detection in the image; and (ii) as based on length measurements of lines of packed droplets. The mean droplet diameters were obtained from the scanning probe image processor elaboration for each acquired image. The mean droplet diameter measurement uncertainty was calculated according to [2].

The autocorrelation and unit cell detection functions were used to enhance periodic structures and to extract the correlation length parameters. The advantage of measuring the mean diameters of lines of packed droplets is that the uncertainty due to border determination is only in the first and last droplets of the line.

Validation of manual and automatic methods through the reference method

The use of the profilometer and scanning probe image processor software image elaboration is a primary reference method for length measurements with known uncertainties. To validate the droplet diameter obtained with the manual and automatic droplet measurement protocols, comparisons with the reference value of the mean droplet diameter obtained by the reference method were carried out. The results of these comparisons were the evaluation of the measurement accuracies of the manual and automatic droplet measurement protocols. The comparisons showed that the both protocols are traceable to the SI through the reference method. The following scheme (scheme M1) shows the traceability chain of the measurement of the diameter of droplets. The first element of the chain is a stabilized laser radiation which is a reference for length. The laser radiation in combination with a diffractometer (instrument able to analyse the structure of a material from the scattering pattern produced when a light radiation interacts with it) is used to measure distances within a 2D grating (a grid with nominal distance between regularly spaced elements). The 2D calibrated grating is used as reference for calibrating distances in images produced with the optical profilometer. The optical profilometer is finally used to measure droplet diameter as reference method.

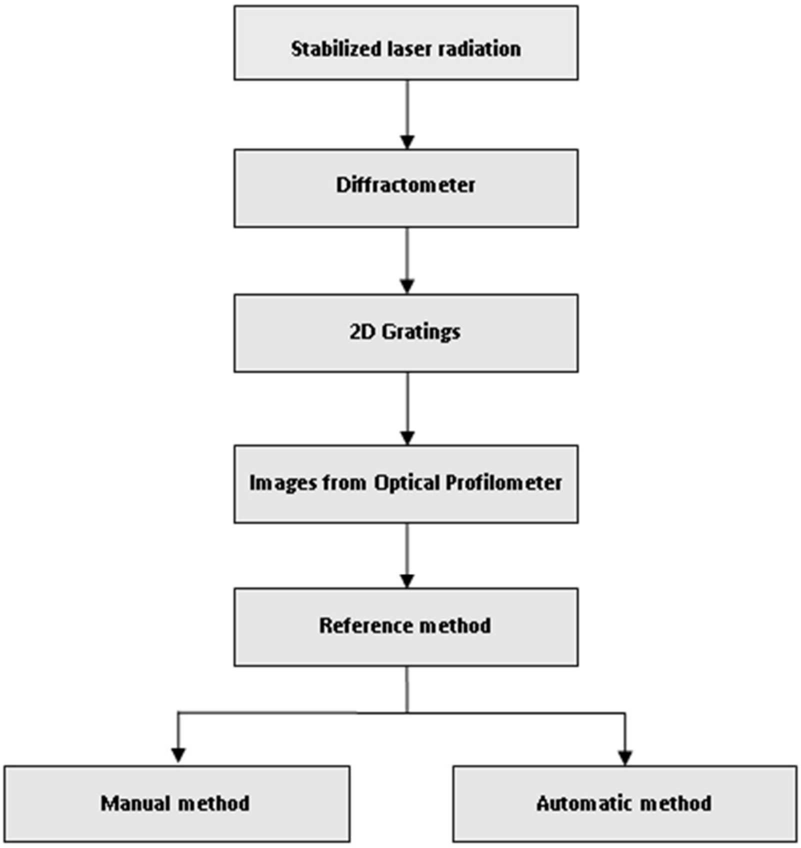

Scheme M1 Traceability chain of droplet diameter measurement

Methods S2: Manual image analysis

Briefly, the Image J™ [3, 4] segmented line tool was used to measure the distance between the first and the last droplets of a line, which were then divided by the number of droplets to calculate the mean diameter. From the mean diameter, the volume was calculated after calibrating the pixel size using a certified micrometer stage. A detailed description is provided below.

The manual analysis of droplet images required: (i) measurement of the length scale of the microscope, by measurement of the pixel dimension with an X-Y ruler (or calibration grid) (Fig. M1); and (ii) measurement of the droplet diameter on lines of droplets that were touching each other (Fig. M2). These images were acquired at 10× magnification, and the analysis was carried out only on lines of droplets that were self-packed within each image. The X-Y ruler used by INRiM had nominal X and Y pitch scales of 25 µm, and additionally, it had been calibrated by the INRiM profilometer. The ruler calibration uncertainty was 0.15%.

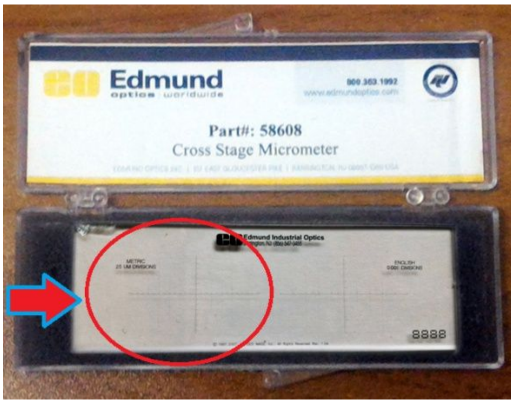

Fig M1 XY ruler with a cross on the metric scale (left, circled in red) and a cross on the inch scale (right)

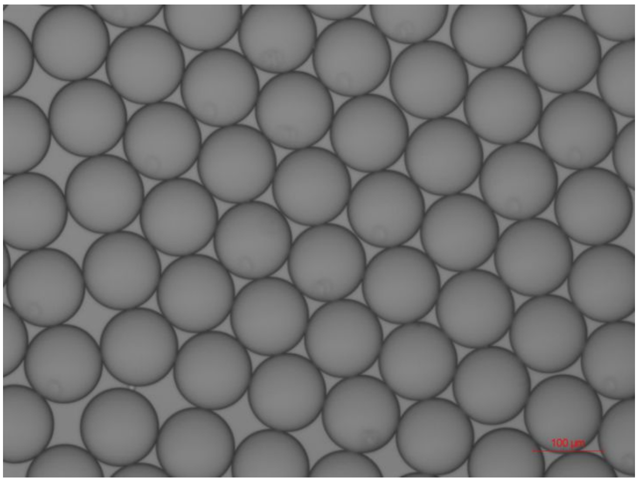

Fig M2 Lines of self-packed droplets

__Pixel dimensions measurement__

A calibrated XY micrometer (or ruler) was used for the pixel dimensions as follows:

1. Position the XY calibrated ruler on the microscope stage.
2. Set the microscope for phase contrast imaging and the same magnification that is to be used for acquiring the droplets.
3. Place the metric scale of the ruler in the centre of the camera view.
4. Adjust the z-axis to focus the XY ruler scale and set the exposure time to achieve the optimum optical contrast (Fig. M3, red square shows the minimum image portion to acquire).

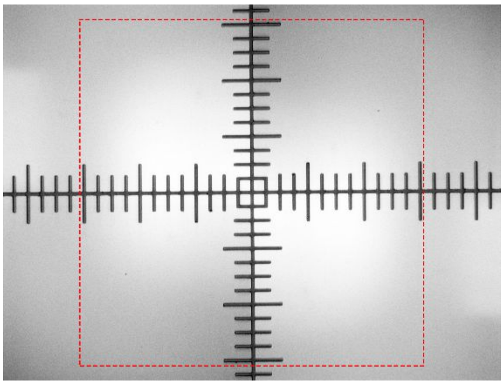

Fig M3 Image of the XY ruler portion for the acquisition

5. Acquire the image of the cross in the metric scale so that at least 600 µm × 600 µm (Fig. M3, red square) is visualised, and save this; the dimension of this square is required to have a statistically sufficient number of pixels (the two certified segments are less than the side of the red square).
6. With Image J^{TM} (version 1.50a used here), open the cross image and select the tool 'Rectangular' on the software main window (Fig. M4).

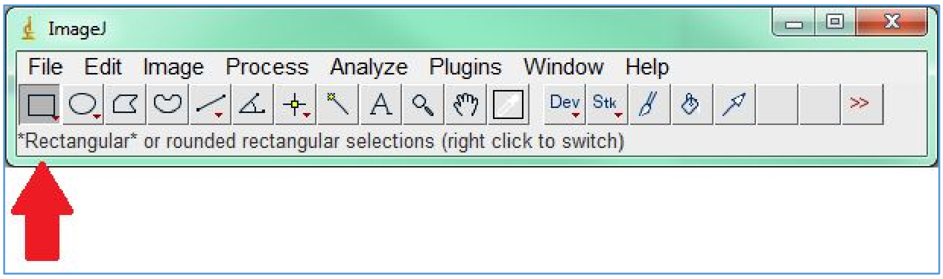

Fig M4 Image J 'Rectangular' tool

7. Use the ‘Rectangular’ tool to select the portion of the image, and measure the number of pixels on the x and y axes of that portion (pixel numbers of X axis and Y axis correspond to the two certified segments).
8. Draw a square as close as possible to the area that includes the certified segments (Fig. M5).

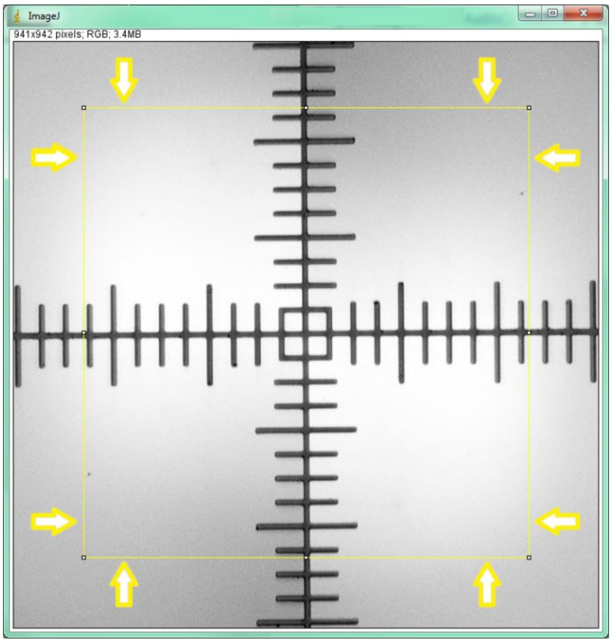

Fig M5 Square drawn around the portion of the XY ruler for the acquisition

9. After this coarse selection, refine the selection of the square using the ‘+’ key of the keyboard; zoom-in on the image while keeping the mouse indicator on the centre of the view to zoom (Fig. M6).

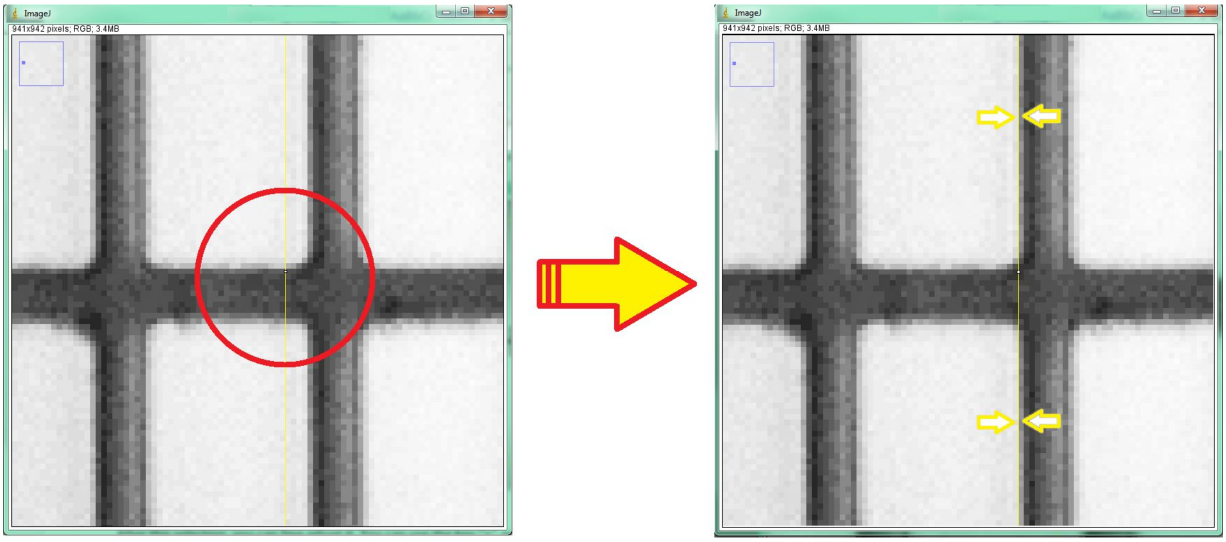

Fig M6 Left side of the cross metric scale (-225 μm)

10. To resize the yellow square, go on the little white square on the yellow line (Fig. M6, red circle; the mouse indicator will change from an arrow 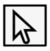 to a hand 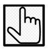 when it is possible to drag the line) and drag the line to the end of the black line (Fig. M6, on the left side of the nominal -225 μm).

11. Repeat point 9 for all of the other square sides. When the yellow square is resized, crop the portion of the image (Fig. M7).

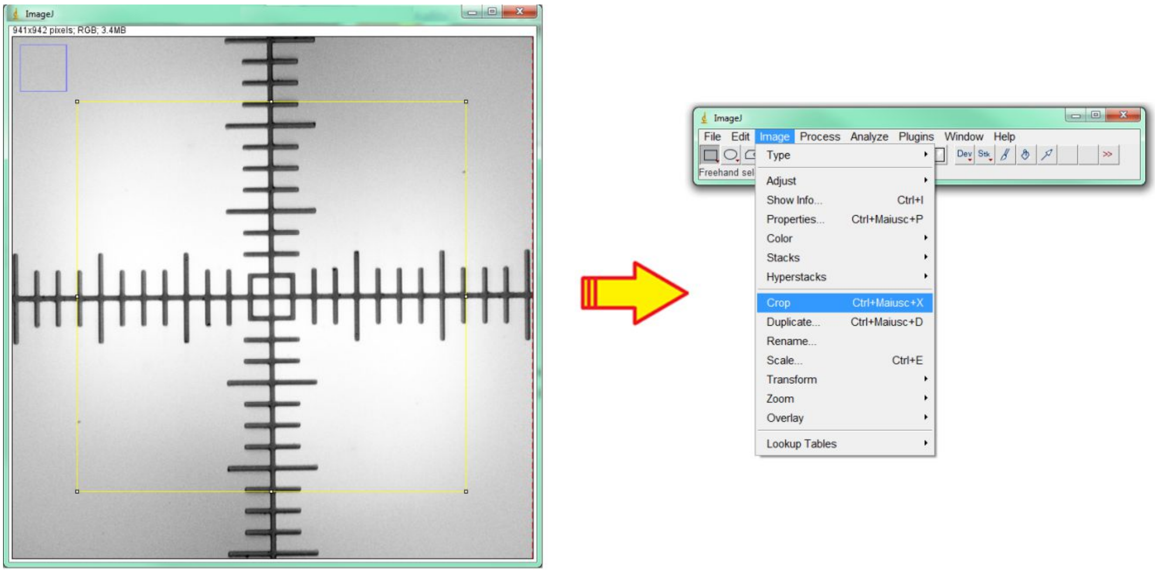

Fig M7 Crop the image

11. The number of pixels will appear at the top left of the image windows (Fig M8); the first value is the number of pixel for the horizontal axis (x), and the second for the vertical axis (y).

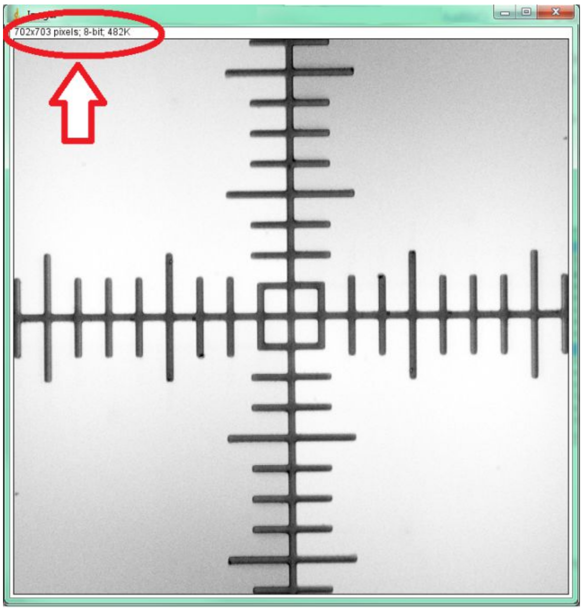

Fig M8 Number of pixels shown at the top left corner

12. Calculate the pixel dimension (D) in micrometres, for both the horizontal (x axis) and the vertical (y axis) scales using Equation (M1):

$$D = \frac{L}{N}\ (\mu\text{m}) \qquad \text{(M1)},$$

where N is the number of pixels in the segment considered, and L is its length in micrometres (the nominal value of this length is -225 μm to +225 μm; considering the external edge of the scale lines, the total length (L) measured is x = 458 ±0.7 μm and y = 458 ±0.7 μm; Fig. M8).

13. The pixel dimensions (D) in micrometres for both the horizontal and vertical scales given were calculated as:

D = 458 μm/ 702 pixel = 0.652 μm/pixel → pixel width (x)

D = 458 μm/ 703 pixel = 0.651 μm/pixel → pixel height (y)

It is possible to assume that each pixel is a square with 0.65 μm per side, and to express the droplet diameters in micrometres (and not only in pixels).

Droplet diameter measurement

The manual droplet measurement protocol is described here.

1. Open ImageJ™ and an image showing lines of self-packed droplets (Fig. M9).

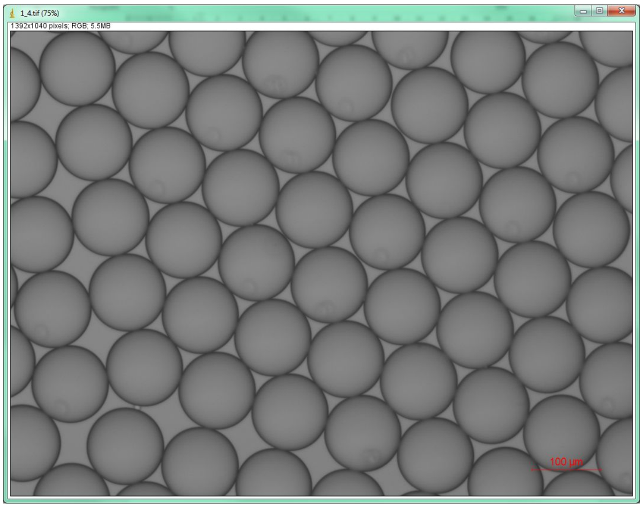

Fig M9 Droplets forming lines imaged at 10× magnification

2. Right click on the 'Straight line' tool (Fig. M10).

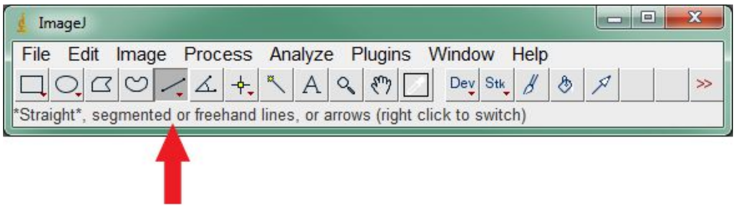

Fig M10 The 'Straight line' tool in Image J™

3. Left click to select the 'Segmented line' tool (Fig. M11). The tool icon will change. With this tool it is possible to draw a line on the droplets. Left click to set the 'beginning', and right click to set the 'end' of the line selection.

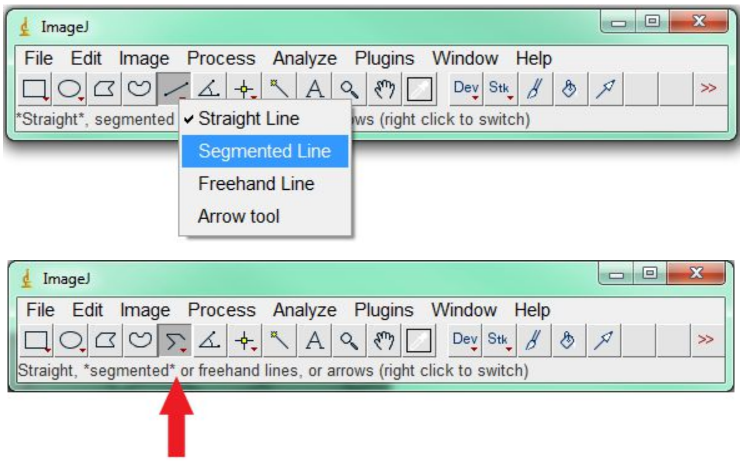

Fig M11 The 'Segmented line' tool in Image J™

4. It is recommended to use the zoom function ('+' and '–' of the keyboard) for accurate drawing of the segmented line. Zoom in at the edge of the first droplet (move the mouse cursor onto the portion of interest and press the '+' key). Left click on the edge to set the line 'beginning' (Fig. M12).

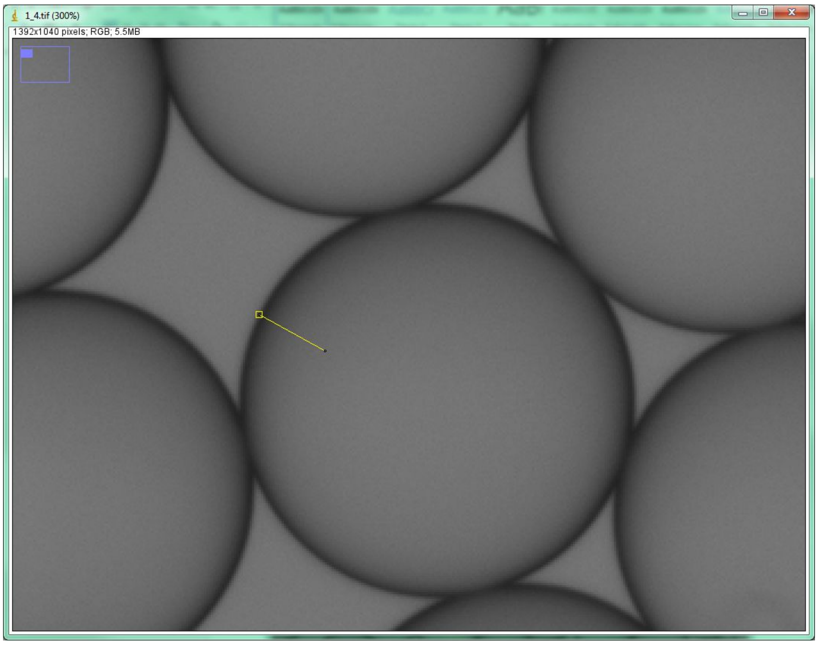

Fig M12 Setting the line beginning

5. Zoom out (using the '–' key) to see the entire zone again, and move the cursor to the external edge of the last droplet to be included in the selection (Fig. M13).

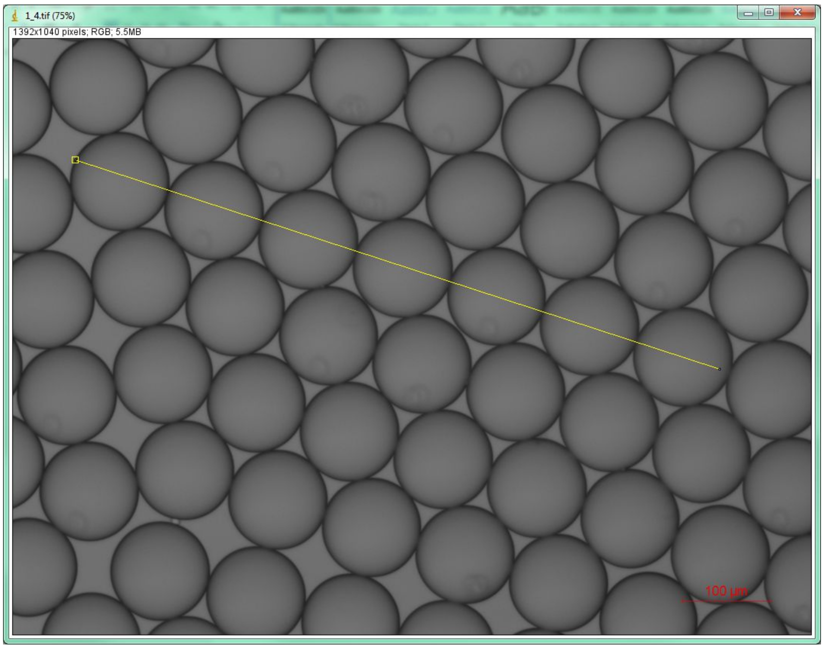

Fig M13 Selecting the last droplet

6. Zoom in around the external edge of the last droplet, and right click on the external edge to select the end of the line of the self-packed droplets (Fig. M14).

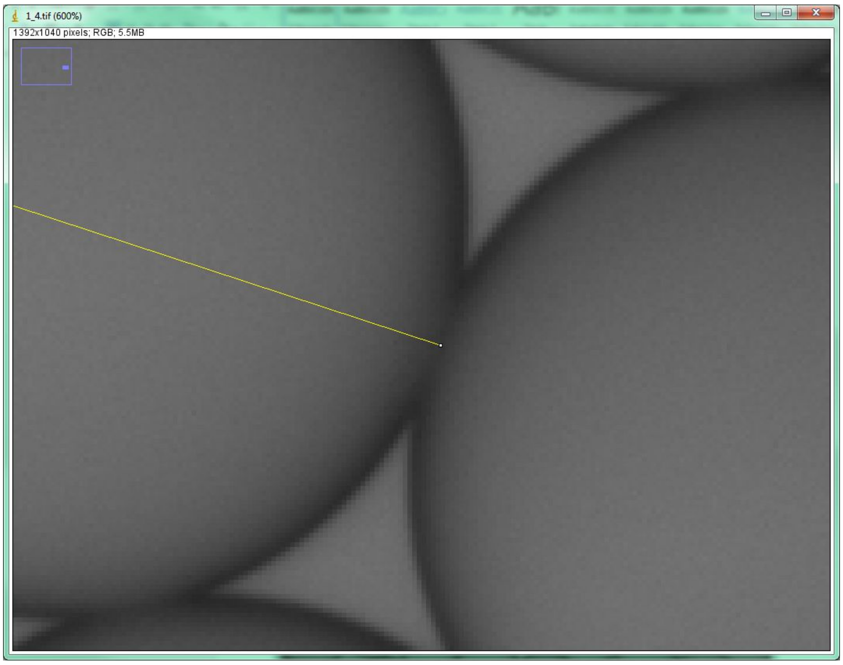

Fig M14 Setting the end of the line

7. Zoom out until the entire image can be seen, to check the line of the selected droplets (Fig. M15).

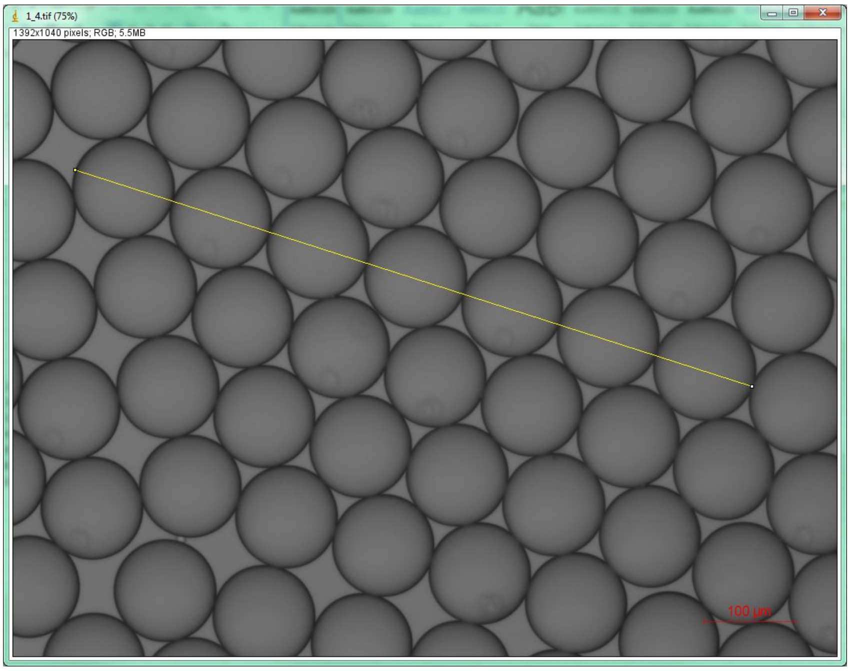

Fig M15 The selected line of droplets

8. On the main ImageJ™ window, click on 'Analyze → Plot Profile' (alternatively, use the keyboard shortcut of 'CTRL + K') (Fig. M16).

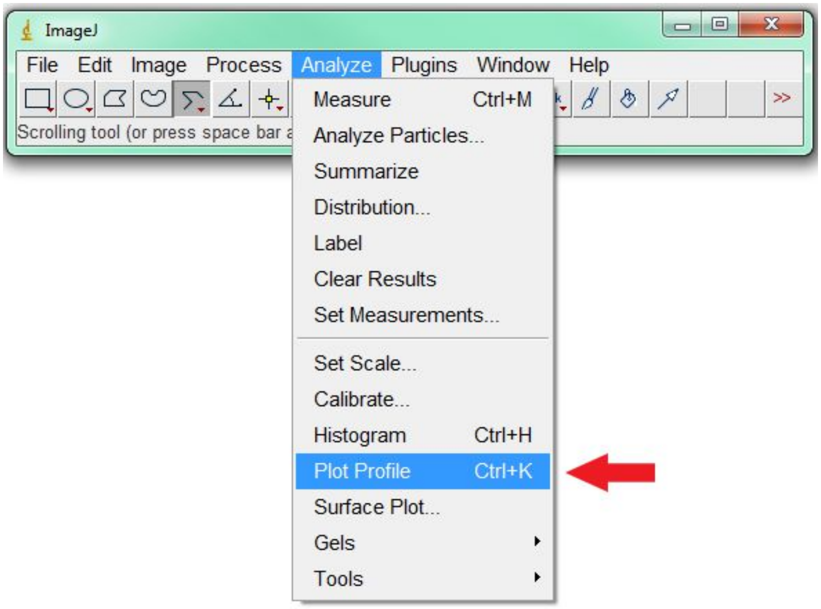

Fig M16 Selecting 'Plot Profile'

9. A new window will open with the results of the line selection: the number of peaks in the 'Plot' window represents the number of droplets in the selection (Fig. M17).

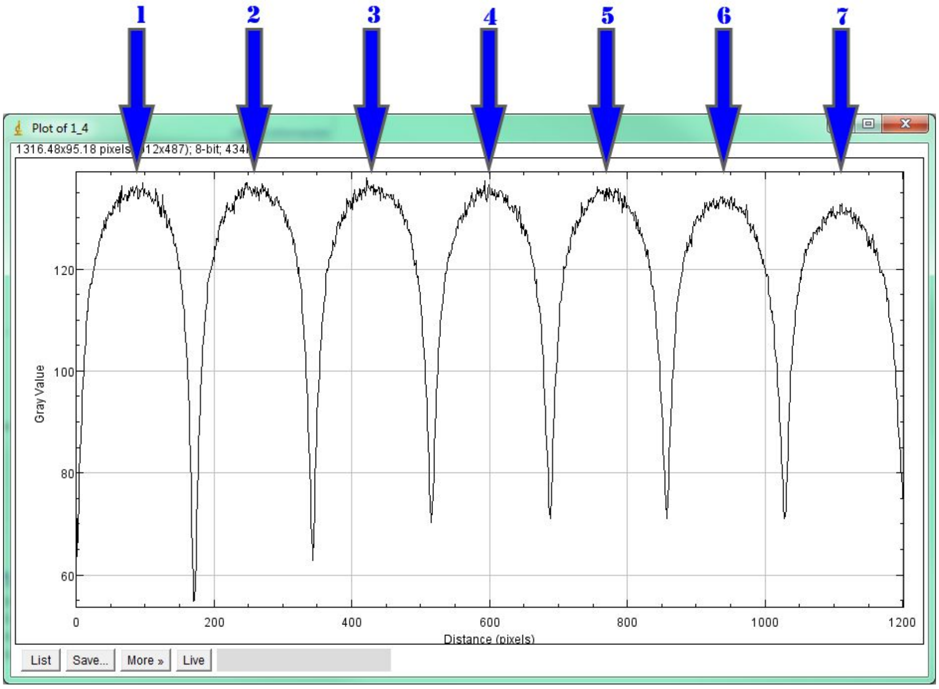

Fig M17 The 'Plot' window

10. Click on 'List' (bottom left) to see the list of Plot values (Fig. M18).

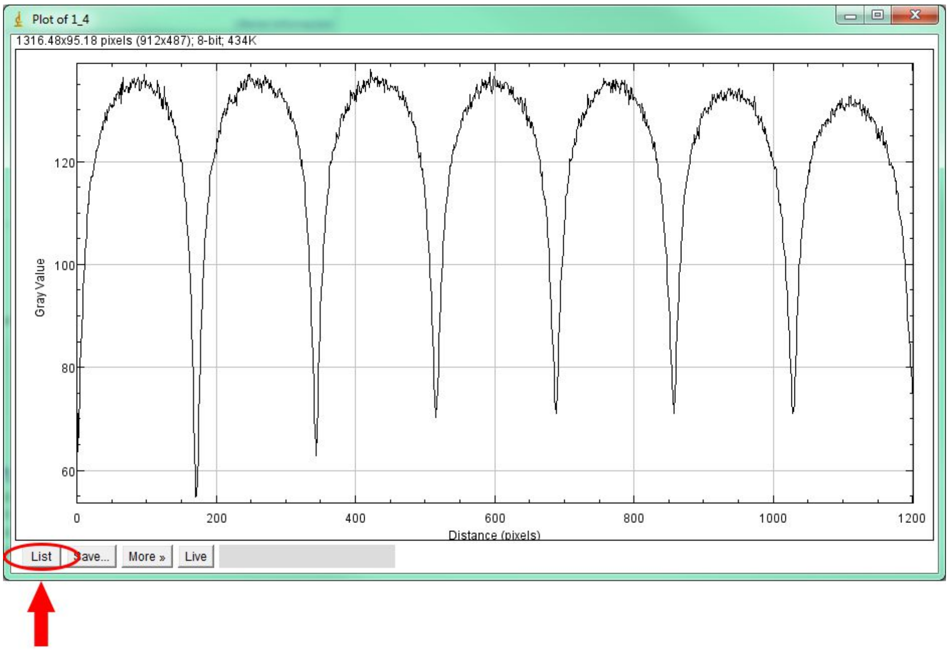

Fig M18 Click on 'List'

11. The last plot value of the list is the total length (in pixels) of the line of droplets selected (Fig. M19).

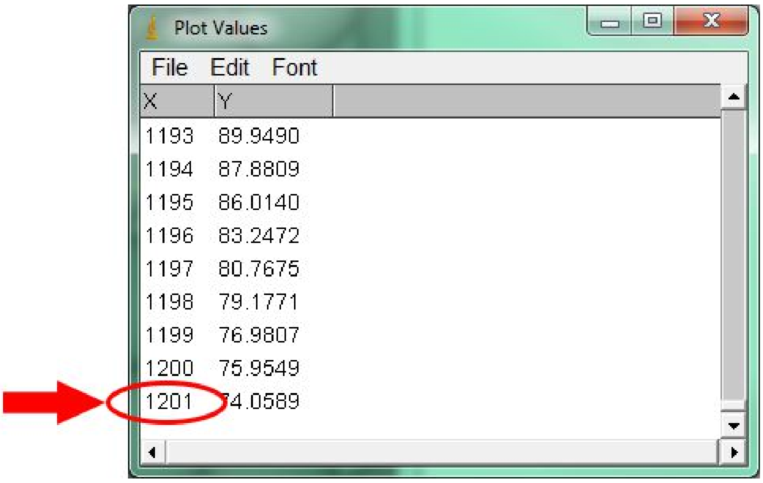

Fig M19 Total length of line of droplets selected

12. Calculate the mean droplet diameter using Equation M2:

$$\textit{Mean droplet diameter} = \frac{\textit{Length of droplets line}}{\textit{Number of droplets}}\,[\text{pixels}] \qquad \text{(M2).}$$

Then convert the mean diameter in pixels to the mean diameter in µm, using the known pixel dimension.

Example:

Length of droplet line: 1201 pixels

Number of droplets: 7

Pixel dimension: 0.65 µm

$$\textit{Mean droplet diameter}\ [\textit{pixels}] = \frac{1201\ \textit{pixels}}{7} = 171.6\ \textit{pixels}$$

$$\textit{Mean droplet diameter}\ [\mu\text{m}] = 171.6\ \textit{pixels} \times 0.65\ {}^{\mu\text{m}}/_{\textit{pixel}} = 111.54\ \mu\text{m}$$

13. Calculate the mean droplet volume using Equation M3

$$\textit{Mean droplet volume} = \frac{4}{3}\pi r^3 \quad [\mu\text{m}^3] \qquad \text{(M3).}$$

Then convert the mean droplet volume in μm^3 to the mean droplet volume in nL.

Example:

Droplet radius: 111.54 μm/ 2 = 55.77 μm

$$Mean\ droplet\ volume\ [\mu m^3] = \frac{4}{3}\pi \times 55.77^3 = 726592$$

$Mean\ droplet\ volume\ [nL] = \frac{726592}{10^6}$ **= 0.727Methods S3: Automatic image analysis using the Fiji™ software**

For the automatic analysis, for all of the droplets in one image to be analysed at once, the next 14 steps were followed.

Analysis in ImageJ™

1. Open ImageJ™.
2. Open image (File → Open).
3. 'Set scale' (make sure you tick off 'Global'; Fig. M20).
4. Convert to 8-bit (Image → Type → 8-bit).
5. Identify the droplet edges (Process → Find edges).
6. Reduce the noise (Process → Noise → Despeckle).
7. Specify the width of the edges (Image → Adjust → Threshold).
8. Enable separation of the droplets (Process → Binary → Make binary).
9. Separate touching droplets (Process → Binary → Watershed).
10. Specify the width of the edges again (Process → Binary → Varonoi).
11. Identify the edges again (Image → Adjust → Threshold).
12. Measure (Analyse → Analyse Particles).
13. Set the parameters to only measure objects with circularity greater than 0.85 (Fig. M21), and hit OK.
14. A table of measurements, named 'Results', will open. Save this (File → Save as) for further Excel™ analysis. Also save the image after the analysis (Fig. M22), as this will help to determine which measurements to consider in the Excel™ analysis.

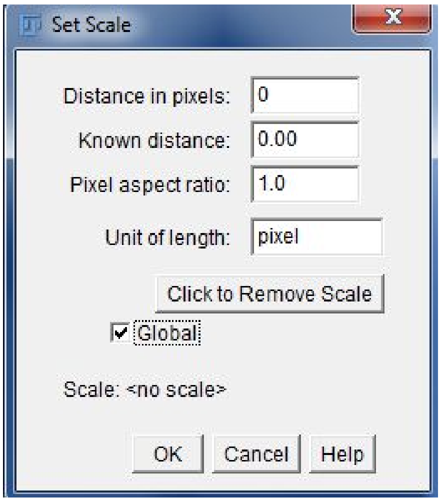

Fig M20 Setting the scale. Make sure to tick off 'Global'

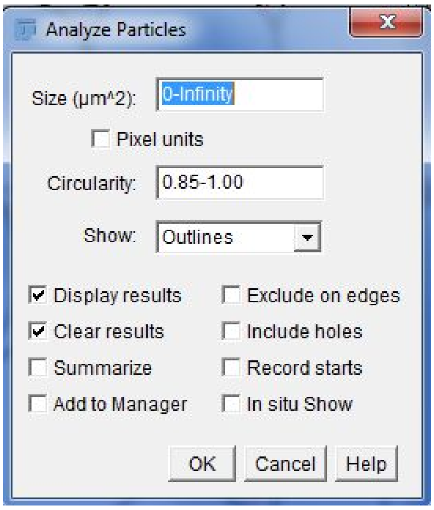

Fig M21 Measurement parameters

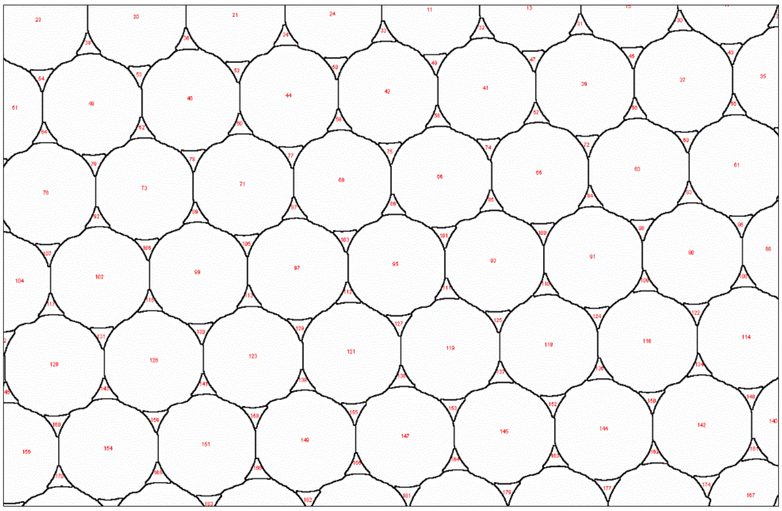

Fig M22 Droplets after the ImageJ^{TM} analysis

Analysis in Excel™

The analysis in ImageJ™ was followed by analysis in Excel™. Figure M23 shows the output in Excel™ format. As there is a possibility that, even though the circularity has been set to higher than 0.85, not all measurements correspond to the droplets. To filter out unfit objects only those with roundness (round, column Q) above 0.95 were chosen. Even now all objects may not present the droplets, some objects with a very small area, may be bubbles left from the pipetting. To filter them out, only those measurements that have the area (column B), larger than 6000 (this correspond roughly to a diameter of 80 μm and the volume of 0.27 nL), were chosen. This threshold was chosen because there is an extremely low probability that an object of this size could represent a droplet, and even if this would be an actual droplet it would be excluded by the droplet reader.

A	B	C	D	E	F	G	H	I	J	K	L	M	N	O	P	Q	R
	Area	Mean	Min	Max	Perim.	Major	Minor	Angle	Circ.	Feret	FeretX	FeretY	FeretAngle	MinFeret	AR	Round	Solidity
29	21443	0	0	0	562.985	165.270	165.197	97.243	0.85	172.003	1203	613	169.278	164.420	1.000	1.000	0.973
5	21275	0	0	0	560.357	164.701	164.469	69.623	0.851	171.234	1220	145	144.268	162.441	1.001	0.999	0.972
14	21341	0	0	0	556.257	165.374	164.307	49.407	0.867	171.304	1157	392	27.463	163.838	1.006	0.994	0.977
44	22091	0	0	0	564.742	168.303	167.122	92.625	0.87	173.589	352	978	28.189	166.583	1.007	0.993	0.976
7	21386	0	0	0	557.286	165.695	164.335	127.740	0.865	175.354	1047	262	27.880	163.000	1.008	0.992	0.976
17	21389	0	0	0	550.943	165.696	164.357	13.869	0.885	171.330	995	421	25.219	163.489	1.008	0.992	0.98
19	21783	0	0	0	563.470	167.185	165.894	87.112	0.862	173.727	719	354	113.762	166.039	1.008	0.992	0.973
26	21729	0	0	0	557.428	166.976	165.689	37.221	0.879	171.540	63	635	60.297	165.036	1.008	0.992	0.981
34	21807	0	0	0	566.056	167.409	165.855	86.719	0.855	174.072	799	807	88.354	165.052	1.009	0.991	0.975
37	23417	0	0	0	588.056	173.458	171.888	35.039	0.851	180.878	1141	775	171.736	171.141	1.009	0.991	0.973
40	22245	0	0	0	565.227	169.027	167.566	70.342	0.875	173.566	832	882	25.236	166.180	1.009	0.991	0.977
18	21136	0	0	0	543.772	164.861	163.235	88.223	0.898	169.000	836	440	22.620	162.311	1.010	0.99	0.984
27	21967	0	0	0	560.985	168.040	166.444	59.002	0.877	172.569	690	675	85.347	165.673	1.010	0.99	0.98
28	21493	0	0	0	557.227	166.302	164.554	82.396	0.87	171.759	498	545	115.521	163.575	1.011	0.989	0.979
32	20552	0	0	0	542.985	162.695	160.839	62.369	0.876	167.765	203	764	84.527	160.034	1.012	0.989	0.981
35	21956	0	0	0	560.399	168.099	166.302	83.433	0.879	173.528	604	670	115.974	165.721	1.011	0.989	0.979
30	21571	0	0	0	556.642	166.763	164.695	28.481	0.875	170.848	290	644	174.289	163.448	1.013	0.988	0.98
8	21543	0	0	0	560.742	166.716	164.527	116.056	0.861	172.325	203	156	111.801	164.304	1.013	0.987	0.975
45	21317	0	0	0	560.156	165.814	163.687	115.258	0.854	170.988	1097	930	171.254	163.475	1.013	0.987	0.975
23	21817	0	0	0	560.399	167.818	165.526	63.831	0.873	173.046	261	616	83.696	165.627	1.014	0.986	0.979
9	20942	0	0	0	542.701	164.532	162.060	60.623	0.894	170.488	891	282	24.611	162.000	1.015	0.985	0.983

Fig M23 Output of ImageJ™ in Excel™

Every object that was measured has its own number, which is in the first column of the Excel™ file (Fig. M23, column A). If a measurement appears to be an outlier, it is necessary to check if the object measured really corresponds to a droplet.

The object area was calculated using the major and minor axes of the best-fit ellipse according to equation M4. For this reason, only the data for area, major and minor axes, circularity and roundness are left. From this point on, the object is assumed to be a perfect sphere, thus radius and volume have been calculated using equations for a sphere, equations M5 and M6, respectively.

$$A = \frac{minor * major * \pi}{4} \tag{M4}$$,

$$r = \sqrt{\frac{A}{\pi}} \tag{M5}$$,

$$V = \frac{\frac{4}{3} * \pi * r^3}{10^6} \tag{M6}$$.

References

1. Meli F, Klein T, Buhr E, Frase CG, Gleber G, Krumrey M, Duta A, Duta S, Korpelainen V, Bellotti R, Picotto GB, Boyd RD, Cuenat A. Traceable size determination of nanoparticles, a comparison among European metrology institutes. Meas Sci Technol 2012;23:125005. doi: 10.1088/0957-0233/23/12/125005
2. Joint Committee for Guides in Metrology. Evaluation of measurement data - guide to the expression of uncertainty in measurement. 2008.
3. Schindelin J, Rueden CT, Hiner MC, Eliceiri KW. The ImageJ ecosystem: an open platform for biomedical image analysis. Mol Reprod Dev 2015;82:518–29. doi: 10.1002/mrd.22489
4. Schneider CA, Rasband WS, Eliceiri KW. NIH Image to ImageJ: 25 years of image analysis. Nat Meth 2012;9:671–5.
